# Diversity of trion states and substrate effects in the optical properties of an MoS_2_ monolayer

**DOI:** 10.1038/s41467-017-02286-6

**Published:** 2017-12-14

**Authors:** Matthias Drüppel, Thorsten Deilmann, Peter Krüger, Michael Rohlfing

**Affiliations:** 1 Institut für Festkörpertheorie, Westfälische Wilhelms-Universität Münster, 48149 Münster, Germany; 2 Center for Atomic-Scale Materials Design (CAMD), Department of Physics, Technical University of Denmark, DK-2800 Kongens Lyngby, Denmark

## Abstract

Almost all experiments and future applications of transition metal dichalcogenide monolayers rely on a substrate for mechanical stability, which can significantly modify the optical spectra of the monolayer. Doping from the substrate might lead to the domination of the spectra by trions. Here we show by ab initio many-body theory that the negative trion (A^−^) splits into three excitations, with both inter- and intra-valley character, while the positive counterpart (A^+^) consists of only one inter-valley excitation. Furthermore, the substrate enhances the screening, which renormalizes both band gap and exciton as well as the trion-binding energies. We verify that these two effects do not perfectly cancel each other, but lead to red-shifts of the excitation energies for three different substrates ranging from a wide-bandgap semiconductor up to a metal. Our results explain recently found experimental splittings of the lowest trion line as well as excitation red-shifts on substrates.

## Abstract (web summary)

The optical and electrical properties of atomically thin transition metal dichalcogenides critically depend on the underlying substrate. Here, the authors develop an abinitio many-body formalism to investigate the full spectrum of negative and positive trions in these layered semicondutors.

## Introduction

Semiconducting transition metal dichalcogenides (TMDCs) have attracted much attention as candidates for future opto-electronic applications due to their particularly rich physical properties: the indirect-to-direct band-gap transition from bulk to monolayer^1, 2^, selective valley- and spin-excitations^3–7^, and high on–off ratios as field-effect transistors^8^. Because of their two-dimensional nature, most of the measurements of these properties demand a substrate supporting the monolayer. The combination of the two-dimensional structure and enhanced Coulomb interaction causes strong excitonic effects in the monolayers, with reported binding energies of several hundred meV in MoS_2_^9–11^. In addition to excitons, Coulomb interaction can cause the preferred formation of trions, which often dominate the photoluminescence spectra when free charges are present^12–19^. These additional charges can stem from gating, defects, or from a substrate that is used for mechanical stability, a process frequently reported in mechanically exfoliated MoS_2_ monolayers^20, 21^. Depending on the type of the additional charges (electrons or holes) either negative or positive trions will form. While trions have been discussed for quantum dots and similar structures, only few theoretical studies have looked into their properties for TMDCs so far. Experimentally, Ross et al.^13^ used an externally applied voltage that allowed to control the charge of the excitation moving from negative trions to neutral excitons, and further to positive trions. Further studies reveal a splitting of the trion line^22^, as well as asymmetric photoluminescence intensities^22, 23^. In addition, those trions corresponding to the second lowest exciton (B) have been observed^24^. These experimental findings underline the need for a theoretical approach that yields both the complete exciton and trion spectra.

The goal of this work is to address these experimental findings, i.e., to understand the interplay between a TMDC monolayer and a substrate, including the formation and properties of both negative and positive trions and the role of the additional substrate screening quantitatively^25^. For this we apply the framework of ab initio many-body perturbation theory (MBPT, see Supplementary Notes 1 and 2)^26, 27^ and extend it to include trions (Supplementary Note 3). Our computational first-principles approach allows a detailed look into the trion spectra and wave functions in real and reciprocal space, revealing the energetically lowest trion (A^−^) as split into three excitations with both inter- and intra-valley character. Remarkably, the positive trion (A^+^) behaves much different with only one energetically low-lying trion peak. Furthermore, the substrate necessarily contributes dielectric polarizability, and thus weakens the Coulomb interaction inside the TMDC monolayer^28, 29^, causing spectral shifts of both exciton and trion energies as compared to the vacuum environment. It was demonstrated in several works that the screening enhancement causes both strong band-gap renormalization and a reduction of the exciton-binding energy^29–32^. Here we extend the previous findings and verify that these two large effects do not exactly cancel each other, instead they result in small, but robust shifts of both the excitonic and trionic transition energies to the red (lower energies) for TMDC monolayers.

## Results

### Trion excitations

TMDC monolayers often exhibit a second kind of excited state next to excitons: trions^12–19^. They result from the correlation between three excited particles, either two electrons and one hole or one electron and two holes, depending on the type of doping. These additional charges can, for example, be provided by the substrate^20, 21^. Experimentally, their transitions are commonly found at energies slightly below those of excitons. Theoretically, parameter-based descriptions through a model Hamiltonian^33–37^ form an established approach toward these three-particle excitations, predicting binding energies of the A^−^ trion in MoS_2_ of 26–32 meV^38, 39^. Going beyond these approaches, we extend the concepts of MBPT resulting in an ab initio method^40^ that is generally applicable to all systems, overcoming system-specific modeling. We include the full band structure (instead of an effective mass approach) with interband mixing, and as output get the complete trion spectrum, including dark states, and the full atomically resolved trion wave functions in real and **k**-space (for details of the method and numerical convergence see “Methods” section, Supplementary Figs. 1–6 and Supplementary Notes 1–6). Our approach is implemented for both negative and positive trions. Furthermore, we are able to quantify the effect of the substrate screening on trions.

**Fig. 1:**
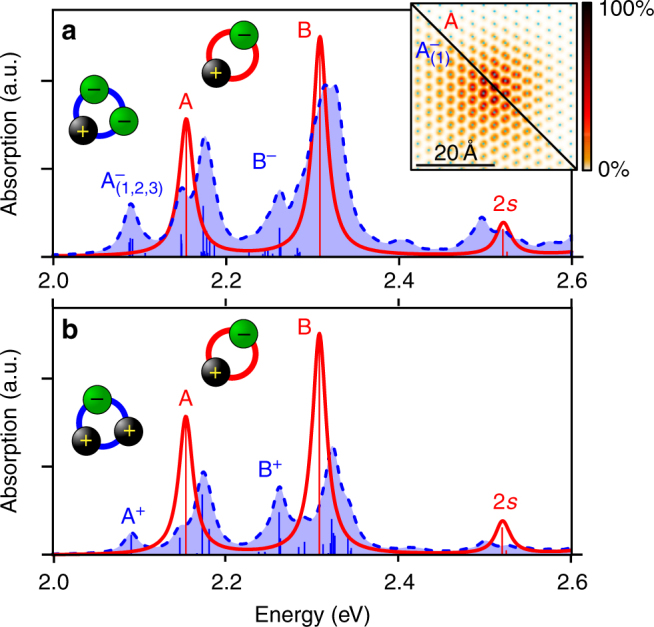
Exciton and trion spectrum of an MoS_2_ monolayer. The excitonic contribution to the optical absorption spectrum is shown in red. The trionic contribution (shown in blue, referring to negative trions in **a** and positive trions in **b**) exhibits resonant states (close to the A and B exciton) and bound states (red-shifted compared to the corresponding excitons). The A^−^ trion is split into three separate peaks, labeled ${\mathrm{A}}_{(1,2,3)}^ -$. The numerical recursion scheme^64^ is used to calculate the energetically lowest states. For the spectra above 2.3 eV in **a** and 2.4 eV in **b**, the Haydock recursion scheme is applied. An artificial broadening of 0.01 eV is used. Inset: comparison between the real space electron distribution (in arbitraty units) of the A exciton with the electron distribution from the ${\mathrm{A}}_{(1)}^ -$ trion (see Supplementary Fig. 7 for details). For this, the hole is fixed in the center of the panel close to a Mo atom, in which the distribution is shown. For the trion state, the distribution of one electron is shown after complete spacial averaging over the second electron

Figure 1 shows both the absorption spectrum derived from first principles due to excitons in red (showing the prominent A and B excitons as well as the A_2*s*_ exciton) and due to trions in blue for a MoS_2_ monolayer as a representative for the whole class of TMDCs. We note that the A_2*s*_ exciton is not completely converged (Supplementary Fig. 4). The first point that is striking in both trion spectra (negative trions in a and positive trions in b) is the huge variety of excitations compared to only two excitons below 2.4 eV. Resonant trions are found close to the A and B excitons, accompanied by bound trions that lie red-shifted below the excitons (A^−^, A^+^ and B^−^, B^+^). Interestingly, the A^−^ trion is split by 1 and 4 meV into three separate peaks, ${\mathrm{A}}_{(1,2,3)}^ -$ (for its detailed analysis see below). The trion-binding energy (in vacuum) is found to be 43 meV for ${\mathrm{A}}_{(1)}^ -$ and 23 meV for B^−^ as well as slightly lower for A^+^ (39 meV) and B^+^ (23 meV). Comparing the absorption intensities, we find that the negative A^−^ trion absorbs more light than the positive A^+^ trion. This is a direct consequence of the three peaks that form A^−^ compared to only one in A^+^. We note that both results, the lower trion-binding energy of A^+^ and the lower intensity are observed experimentally by Jones et al.^22^ for WSe_2_, for which we expect similar results as for MoS_2_. Note that while we are able to compare the intensity of the trions relative to each other, the ratio between trion intensity and exciton intensity depends on the doping. As this is not explicitly taken into account in our calculation, we are not able to directly compare trion to exciton intensities in the spectra.

### Trion wave function and band composition

The modulus squared of the real space wave function of the first bright negative-trion peak ${\mathrm{A}}_1^ -$ is plotted in the inset of Fig. 1a along with the respective distribution for the A exciton. For both, the hole is fixed at the same position close to a Mo atom, and one electron of the trion is spatially integrated out. The result is an insight into the real-space distribution of the trion state, which is much widely extended than the corresponding exciton. We find the root-mean-square radius of the exciton distribution to be $\sqrt {\left\langle {{\mathbf{r}}^2} \right\rangle } = 8.8$ Å, while the corresponding value of the trion distribution is ~30% larger at 11.4 Å. This is a direct consequence of the additional repulsive force between the two electrons involved in the trion state.

**Fig. 2:**
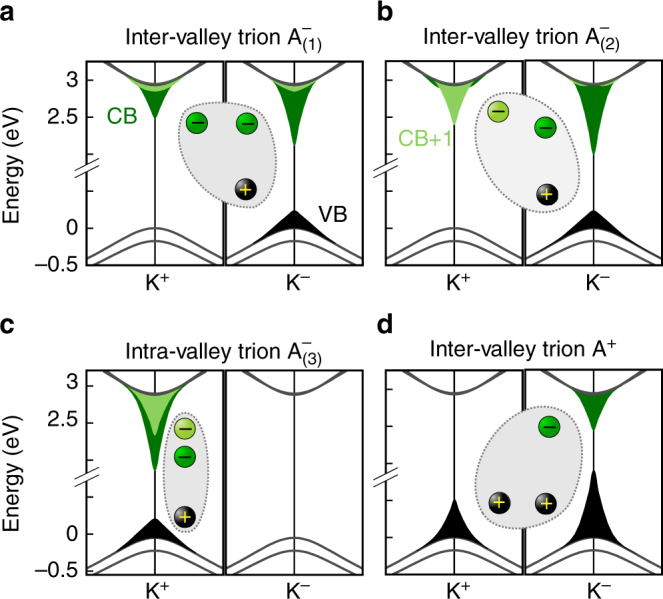
Quasiparticle band structure of an MoS_2_ monolayer. The figure focuses on the vicinity of the K^+^ and K^−^ points. **a**–**c** For all three negative trion excitations ${\mathrm{A}}_{(1,2,3)}^ -$ the band-resolved **k**-dependent contributions (square of the wave functions) are attached to the respective bands (black for the VB and dark (light) green for the CB (CB+1)). For the ${\mathrm{A}}_{(1,2)}^ -$ trions the electrons are located in different valleys, while for ${\mathrm{A}}_{(3)}^ -$ only one valley contributes. The initial one-electron state resides at K^+^ in the CB. **d** Contribution of the A^+^ trion, the initial hole resides at K^+^. We note that in addition to these trions with total momentum K^+^ the same set of trions with exchanged K^+^ ↔ K^−^ occurs for **K** = K^−^

For a deeper understanding of the ${\mathrm{A}}_{(1,2,3)}^ -$ trions, we investigate their contributions in reciprocal space. Figure 2 shows the quasiparticle band structure of the MoS_2_ monolayer in vacuum around K^+^ and K^−^, including the two highest valence bands (VBs) (spin-split by 175 meV) and the two lowest conduction bands (spin-split by 15 meV). Indicated in the band structure are the contributions of each band to all three ${\mathrm{A}}_{(1,2,3)}^ -$ trions (Fig. 2a–c). These three lowest trions are all build up from the highest VB, and the two lowest conduction bands (CB, CB+1), but are still strikingly different. In the simplified picture of an electron bound to an exciton, the occurrence of three states can be easily understood. As the CB at the K valley is occupied by the electron of the bright exciton, the second electron can only reside in one of the other bands/valleys (Pauli exclusion). In addition to the three states shown here with a trion momentum of **K** = K^+^, the same set of excitations exist with **K** = K^−^ (all contributions are found at the different valley K^+^ ↔ K^−^) summing up to six trions in total. While ${\mathrm{A}}_{(1,2)}^ -$ are located both at K^+^ and K^−^ (inter-valley), ${\mathrm{A}}_{(3)}^ -$ is exclusively located at K^+^ (intra-valley). ${\mathrm{A}}_1^ -$ and ${\mathrm{A}}_2^ -$ can be distinguished by their spin character. While all electron spins point into the same direction for ${\mathrm{A}}_2^ -$, the “additional” electron obtains a reversed spin in ${\mathrm{A}}_1^ -$. The inter-valley trion ${\mathrm{A}}_1^ -$ is the energetically lowest conduction band. This can be understood since splitting the two electrons into two valleys allows them to both reside in the energetically lowest conduction band.

Having discussed the splitting for MoS_2_, we note that it is quantitatively different in TMDCs, which include tungsten. Due to the interchanged character of CB and CB+1, we find that for WSe_2_, ${\mathrm{A}}_2^ -$ is moved 30 meV below ${\mathrm{A}}_1^ -$ and now constitutes the lowest trion state. Experimental confirmation of two different kind of trions and practical usability was shown in recent measurements on WSe_2_ by Jones et al.^41^ and on WS_2_ by Boulesbaa et al.^42^. Yu et al.^43^ offered a qualitative explanation for the different trions. Figure 2d shows the corresponding contributions to the A^+^ trion. The lowest optical active state has inter-valley character, where both holes reside in the topmost VB and the electron in the CB at K^−^. The positive trions with one hole residing in the lower VB are not found in the vicinity of the A^−^ trion due to the larger spin–orbit splitting in the VBs compared to the CBs. By considering all possible trion momenta, we find two low-energy states among the positive trions, while six low-energy states are observed among the negative trions. Further trions (positive and negative) are found at higher energy states.

Finally, we note that the (negative) trion states can be distinguished by combining a spin-polarized doping (e.g., from a magnetized substrate) and the usage of circular polarized light. We expect ${\mathrm{A}}_1^ -$ and ${\mathrm{A}}_3^ -$ (with momentum **K**) to be found with one light polarization, while ${\mathrm{A}}_2^ -$ (momentum −**K**) may show up with light of the other polarization. If spin-polarized hole current is used to build up positively charged trions A^+^, we expect them to be only visible with one light polarization while the other will be dark.

### Effects of substrate screening

**Fig.3:**
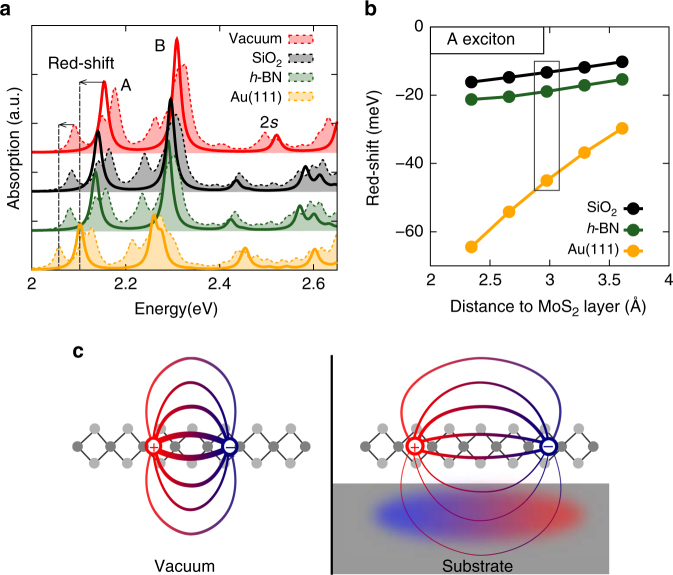
Red-shift of optical transitions due to substrate screening. **a** Exciton (solid lines) and negative trion (dashed and filled lines) absorption spectra of an MoS_2_ monolayer in vacuum and on different substrates. Both spectra stem from a single calculation. The arrows indicate the red-shift in going from MoS_2_ in vacuum to a gold substrate. All substrates are placed at a distance of 3 Å and have a well-converged thickness of about 10 Å (i.e., increasing the substrate thickness by another layer of ~2 Å leads to an excitation shift of less than 0.5 meV). An artificial broadening of 0.01 eV is used. **b** Red-shift of the A exciton energy as a function of the distance to the substrate (using extrapolated values of *N* → ∞ in the **k**-mesh). The box marks the distance used in **a**. **c** Schematically illustration of a bound electron–hole pair in vacuum and on a substrate, where the Coulomb interaction is screened and the substrate is polarized

A substrate might not only donate additional charges, but also drastically enhance the screening environment, which can be captured within our approach. The key results are summarized in Fig. 3a, which compares both the exciton and the negative trion spectra for vacuum, an SiO_2_, a *h*-BN, and a gold substrate. Compared to the free-standing monolayer in vacuum, both the excitons and the trions are red-shifted toward lower energy. Since negative doping through a substrate is frequently reported in TMDCs, we concentrate on negative trions for the substrate effects. The red-shifts of excitons and trions show two clear features: (i) They increase with the substrate polarizability, e.g., in the sequence vacuum → SiO_2_ → *h*-BN → Au in our study (as illustrated by their dielectric constants of *ε*_∞_ = 3.9 (SiO_2_^44^), 4.95/4.10 ($\varepsilon _\infty ^{||}$/$\varepsilon _\infty ^ \bot$ *h*-BN^45^), and ∞ (Au)). (ii) The red-shift increases with decreasing distance between substrate and monolayer. This trend can be seen in Fig. 3b for the A exciton. The smaller the distance, the stronger the screening which leads to a larger red-shift. At the typical van der Waals distance of ~3 Å, we observe red-shifts for the A exciton of 13 meV on SiO_2_, 19 meV on *h*-BN, and 45 meV on Au(111). Note that due to the involved approximations, the relative accuracy (as difference between two values) of our calculations is better than the absolute accuracy. Thus, red-shifts and trion-binding energies are given in meV (see also Table 1). Notably, the excitons experience a stronger shift than the trions, leading to a substrate-dependent trion-binding energy.

**Table 1:** Energy composition of the A exciton and trions

(eV/meV)		A exciton		${\bf{A}}_{\mathbf{(1)}}^{\mathbf{ -}}$ trion		A^+^ trion	
	*E*_g_	Ω^A^	${\boldsymbol{E}}_{\bf{b}}^{\bf{A}}$	${\mathbf{\Omega }}^{{\bf{A}}^{ \mathbf{-}} }$	${\boldsymbol{E}}_{\bf{b}}^{{\bf{A}}^{\mathbf{-}} }$	${\mathbf{\Omega }}^{{\bf{A}}^{\mathbf{+}} }$	${\boldsymbol{E}}_{\bf{b}}^{{\bf{A}}^{\mathbf{+}} }$
Vac.	2.89	2.13	0.76	2.08	43	2.09	39
SiO_2_	2.75	2.12	0.63	2.08	35	2.08	32
*h*-BN	2.73	2.11	0.62	2.07	34	2.08	29
Au	2.25	2.08	0.17	2.05	23	2.06	21

For the A exciton and the ${\mathrm{A}}_{(1)}^ -$ and A^+^ trions on different substrates, we give the bandgap *E*_g_, transition energies Ω, binding energy defined as $E_{\mathrm{b}}^{\mathrm{A}} = E_{\mathrm{g}} - {{\Omega }}^{\mathrm{A}}$ and $E_{\mathrm{b}}^{{\mathrm{A}}_{(1)}^ - } = {{\Omega }}^{\mathrm{A}} - {{\Omega }}^{{\mathrm{A}}_{(1)}^ - }$. All given energies are extrapolated for an infinite supercell size and an infinite **k**-space coverage for the BSE **k**-points, i.e., *N* → ∞ in *N* × *N* × 1. Trion-binding energies are given in meV, all other values in eV.

**Fig. 4:**
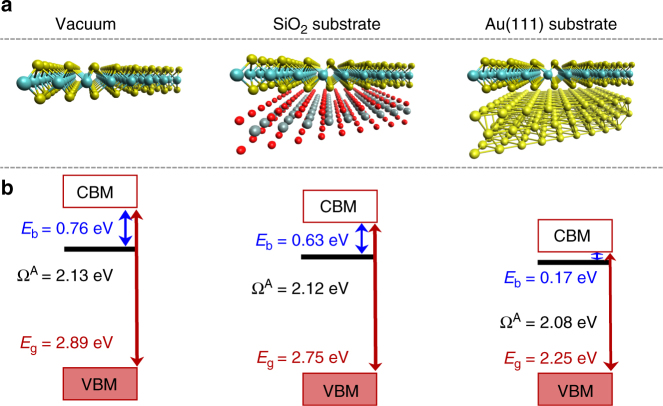
Energy composition of the A exciton on various substrates. We consider vacuum, an SiO_2_ and an Au(111) substrate at a distance of 3 Å (see **a**). For each system the bandgap *E*_g_, the excitation energy Ω^A^ and the binding energy *E*_b_ are given in **b**

The usage of the atom-resolved model function of the LDA+GdW approach^46^ allows a straightforward handling of the screening due to a substrate by including substrate atoms in the dielectric function (Supplementary Note 1). The used method was already successfully employed to describe excitons in WSe_2_ monolayers^47^, and to include the screening of substrates for molecular adsorbates^48, 49^. Note that in this approach the atoms from the substrate change the screening, but do not interact via chemical bonding with the monolayer. The role of screening has already been investigated in parameter-based^39^ and parameter-free^29, 50^ approaches. Comparing vacuum and an SiO_2_ substrate, Kylanpaa et al. have found a difference of 0.18 eV in exciton binding while we found 0.13 eV (Fig. 4). Due to the different inclusion of the substrate (homogenous vs. atomically structured) such a small quantitative difference is not astonishing. We underline that ref. ^39^ does not consider the concomitant change of the band-structure energies. Thus, they do not obtain the combined red-shift. Furthermore, some models might not be directly applicable to cases of metallic screening^39^.

There are a couple of experimental observations on exciton red-shifts in MoS_2_ monolayers. Scheuschner et al.^19^ measured the A^−^ trion in a free-standing monolayer, as well as, on an SiO_2_ substrate. The two data sets of Figure 8 of ref. ^19^ seem to indicate a red shift of about 25 meV. In a recent work by Klein et al.^51^ an MoS_2_ monolayer on a flat surface was subsequently covered by Al_2_O_3_. Comparison of the A exciton without and with the cover shows a red-shift of about 15 meV due to the Al_2_O_3_ cover. In a further experiment, Lippert et al.^52^ found a red-shift of the A exciton of a WSe_2_ monolayer of 12 meV in changing substrates from SiO_2_ to the stronger screening of a sapphire substrate.

### Red-shift of optical excitations

To understand the spectral shifts in the absorption spectra (Fig. 3) we first focus on the excitons. Our calculations allow to identify the exciton red-shifts as a combination of two effects, which are summarized in Fig. 4a, b. First, the substrate renormalizes the fundamental bandgap of the MoS_2_ monolayer. Starting from *E*_g_ = 2.89 eV in vacuum, e.g., an Au(111) substrate reduce the gap by 0.64 to 2.25 eV. Bruix et al.^53^ found a similarly small gap of 1.95 eV for MoS_2_ on Au(111), clearly demonstrating the drastic band-gap renormalization (see also refs. ^29, 54, 55^). The physical origin of the reduction of the bandgap is the image charge effect^50, 56^. The energy needed to introduce an additional electron into the system defines the conduction band minimum (CBM). If a substrate is present, the additional electron will contribute to its polarization, and thus reduce the total energy, shifting the CBM down. The opposite effect holds for the valence band maximum (VBM), thus closing the fundamental gap. Secondly, the exciton binding energy *E*_b_ is reduced: Additional polarizability in the substrate reduces the Coulomb interaction between electron and hole (schematically depicted in Fig. 3c). In the extreme case of the metallic substrate Au(111), the resulting exciton binding energy is 0.17 eV, which is only one-fifth of the vacuum value (0.76 eV). The reduced binding energy is directly visible in the exciton wave functions that gets more extended in real space. We find $\sqrt {\left\langle {{\bf{r}}^2} \right\rangle } = (8.8{\mathrm{/}}9.5{\mathrm{/}}11.2)$ Å for vacuum/SiO_2_/Au(111), respectively (Supplementary Fig. 7).

As a result of our study, we verified that these two large effects (the reduced binding energy and the closing of the gap) do not perfectly cancel each other, but lead to shifts in the excitation energies always to the red (lower energy) for physisorbed systems. Note that we expect that the physisorbed system does not change its structure or occupation when a substrate is introduced. Furthermore, it should not have a long-range static dipole (quadrupole, etc.) moment. A straightforward way of understanding why excitations always shift to the red is to imagine the excited state (and the corresponding total energy) with and without the substrate. The additional degree of freedom due to the surface polarizability will always lower the total energy. Furthermore, the substrate will adjust its charge density, known for metals as image charges^50^. This gained energy leads to a reduced excitation energy, i.e., a red-shift compared to vacuum. Such red-shifts have been reported in many materials^57^ and can be seen as an optical analog to the image-charge effect where a dipole-like image forms as schematically depicted in Fig. 3c. We note that in experiments further (blue or red) shifts might occur due to, e.g., strain or hybridization.

Table 1 compares the bandgap, exciton and trion excitation, and binding energies for the analyzed substrates. As mentioned above, the red-shift of the A exciton is smaller than the red-shift of the A^−^ trion. This results in a trion-binding energy $E_{\mathrm{b}}^{{\mathrm{A}}^ - }$ that is substrate dependent. The corresponding values are also listed for the B^−^ trion showing the same characteristics. On the Au(111) substrate $E_{\mathrm{b}}^{{\mathrm{A}}^ - }$ gets reduced by more than 50%. The binding results from the Coulomb interactions between the hole and the two excited electrons. Weakening this interaction through screening thus leads to a weaker bond between the three particles. For the A^−^ trion on SiO_2_, we find $E_{\mathrm{b}}^{{\mathrm{A}}^ - } = 35$ meV, while the corresponding binding energy of the positive trion is slightly smaller with 32 meV. These values agree very well with experimental results with values between 20–43 meV^12, 13, 58, 59^ (on SiO_2_ substrates). On the other hand, we note that absolute transition energies slightly deviate as discussed earlier^60^. In the case of a WSe_2_ monolayer, a reduction of the trion-binding energy by ~10% was found by Lippert et al.^52^ increasing the screening from an SiO_2_ to a sapphire substrate. Furthermore, we are able to predict a binding energy of the B^−^ trion of 23 meV in vacuum and 18 meV on SiO_2_ (for B^+^ 23 and 17 meV). Experimentally the B^−/+^ trion has been observed by reflectance measurements by Yang et al.^24^ in WSe_2_ and MoSe_2_.

## Discussion

The additional doping by substrates in two-dimensional semiconducting TMDCs frequently results in the formation of trions. Taking an MoS_2_ monolayer as representative, these were addressed by our novel theoretical framework. We could reveal the diversity of trion states with both resonant and bound trions. We find one energetically low, optically active A^+^ trion, while A^−^ shows a fine splitting into three excitations with inter- and intra-valley character. Our results explain recent experimental findings that showed more than one trion excitation. At higher energy, also the B excitons are accompanied by red-shifted B^+/−^ trions. In addition, we have discussed the influence of three widely used substrates (SiO_2_, *h*-BN, and Au(111)). The additional screening renormalizes both the electronic gap, the exciton, and trion-binding energies. For the three investigated substrates, we find that the effects on the exciton and trion excitation energies do not exactly cancel each other, such that a shift always to lower energy is left. The trion-binding energies for the A^−^ (A^+^) trion of ~43 meV (~39 meV) in vacuum are slightly reduced on an SiO_2_ substrate to ~35 meV (~32 meV). Our quantitative results concerning the binding energies as well as the magnitude of the A exciton red-shifts are in good agreement with experimental observations. Our findings are especially relevant since most possible opto-electronic applications involve substrates, which makes a deeper qualitative and quantitative understanding of the changes induced by the drastically enhanced screening environment essential both for excitons and trions.

## Methods

### Bethe–Salpeter equation for excitons

For the excitonic absorption spectra in Fig. 1 we solve the Bethe–Salpeter equation (BSE), starting from a preceding GW calculation within MBPT^61, 62^ (see also Supplementary Notes 1 and 2). In the self-energy operator of the GW, we employ the simplified LDA + GdW approximation^46^ for numerical efficiency. Here, the dielectric screening properties are described by an atom-resolved model function based on the random-phase approximation^46^. The LDA + GdW approximation yields good results for the MoS_2_ monolayer at a fraction of the numerical cost of a GW calculation, which is, e.g., used by Qiu et al.^60^ who find the excitation energy of the A exciton as Ω^A^ = 2.04 eV and Ω^B^ = 2.17 eV. Our approach yields Ω^A^ = 2.13 eV and Ω^B^ = 2.29 eV.

### Equation of motion for trions

The presented trion spectra and wave functions are obtained as eigenstates of an effective Hamiltonian (for negative trions) with matrix elements^40^

1 $$\begin{array}{*{20}{l}}{\left\langle {{\bf{vc}}_1{\bf{c}}_2\left| {\hat H^{({\mathrm{eeh}})}} \right|{\bf{v}\prime\bf{c}}_1\prime {\bf{c}}_2\prime } \right\rangle } \hfill & \hskip-8pt = \hfill &\hskip-7pt{\left( {\epsilon _{{\bf{c}}_1} + \epsilon _{{\bf{c}}_2} - \epsilon _{\bf{v}}} \right)\delta _{{\bf{c}}_1,{\bf{c}}_1\prime }\delta _{{\bf{c}}_2,{\bf{c}}_2\prime }\delta _{{\bf{v}},{\bf{v}\prime}}\qquad \left( {\hat H_{{\mathrm{BS}}}} \right)} \hfill \\ {} \hfill & {} \hfill & { + \left( {W_{{\bf{c}}_1{\bf{c}}_2,{\bf{c}}_1\prime {\bf{c}}_2\prime } - W_{{\bf{c}}_1{\bf{c}}_2,{\bf{c}}_2\prime {\bf{c}}_1\prime }} \right)\delta _{{\bf{v}},{\bf{v}\prime}}\quad \left( {\hat H_{{\mathrm{ee}}}} \right)} \hfill \\ {} \hfill & {} \hfill & { - \left( {W_{{\bf{v}\prime c}_1,{\bf{vc}}_1\prime } - V_{{\bf{v}\prime c}_1,{\bf{c}}_1\prime {\bf{v}}}} \right)\delta _{{\bf{c}}_2,{\bf{c}}_{2}\prime}\qquad \left( {\hat H_{{\mathrm{eh,1}}}} \right)} \hfill \\ {} \hfill & {} \hfill & { - \left( {W_{{\bf{v}\prime c}_2,{\bf{vc}}_2\prime } - V_{{\bf{v}\prime c}_2,{\bf{c}}_2\prime {\bf{v}}}} \right)\delta _{{\bf{c}}_1,{\bf{c}}_1\prime }\qquad \left( {\hat H_{{\mathrm{eh,2}}}} \right)} \hfill \end{array}$$

(see also Supplementary Note 3). Here the short-hand notation **v** = (*v*, **k**_*v*_) is used for band index and **k**-vector. $\epsilon _{{\bf{c}}_1}$ etc. denote the quasiparticle band-structure energies, *V*_*i*_ the bare, and *W*_*i*_ the screened Coulomb interaction $W\left( {{\bf{r}},{\bf{r{\prime}}}} \right) = {\int} \varepsilon ^{ - 1}\left( {{\bf{r}},{\bf{r{\prime\prime}}}} \right)V\left( {{\bf{r{\prime\prime}}},{\bf{r{\prime}}}} \right)\mathrm{d}^3{\bf{r{\prime\prime}}}$, where the dielectric function enters. The Hamilton matrix is constructed from four parts: the band structure term $\hat H_{{\mathrm{BS}}}$, electron–electron repulsion $\hat H_{{\mathrm{ee}}}$, and the attraction between the hole and both electrons $\hat H_{{\mathrm{eh}},1/2}$. Each of the last three is split into a direct part and an exchange part of the Coulomb interaction. The total momentum of the trion state is fixed and determines **k**_1_ + **k**_2_ − **k**_*v*_ = **K**. Since the absorbed photon has nearly zero momentum, the total momentum of the trion is given by the momentum of the electron from which the trion state is excited. In the present study, this additional electron can reside in either one of the two lowest conduction bands (split by 15 meV) at the K points of the Brillouin zone (for the **K** = K transition the optical dipoles are found to be by far the strongest). Leaving out the second electron reduces Eq. (1) to the BSE Hamiltonian $\left\langle {{\bf{vc}}\left| {\hat H^{({\mathrm{eh}})}} \right|{\bf{v{\prime}c{\prime}}}} \right\rangle = \left( {\epsilon _{\bf{c}} - \epsilon _{\bf{v}}} \right)\delta _{{\bf{cc{\prime}}}}\delta _{{\bf{vv{\prime}}}} - \left( {W_{{\bf{v{\prime}c}},{\bf{vc{\prime}}}} - V_{{\bf{v{\prime}c}},{\bf{c{\prime}v}}}} \right)$, commonly used for excitons within MBPT^26, 27^. The diagonalization of the trion Hamilton matrix is numerically extremely demanding due to the immense dimension of ~10^7^ for a typical calculation and relies on recursive diagonalization schemes^63, 64^. We apply a 27 × 27 × 1 **k**-space grid in the 2D Brillouin zone and include two valence and four conduction bands. Note that within our approach the A and B excitons and trions converge extremely fast with respect to the **k**-mesh. This is discussed in detail in Supplementary Figs. 3–6. For all calculations presented in this work, a supercell of 45 Å perpendicular to the monolayer is used. Further numerical details of our calculations are discussed in Supplementary Notes 4–6 and Supplementary Figs. 1 and 2. A detailed introduction to the method and the Hamilton matrix can be found in ref. ^40^. We note that we do not include changes of the initial band structure due to additional carriers^65^, i.e., the presented spectra describe the limit of very-low doping. Positive trions can be described by the reformulation of Eq. (1) in which one of the excited electrons is replaced by an excited hole.

### Data availability

The data that support the findings of this study are available from the corresponding author upon reasonable request.

## Electronic supplementary material

Supplementary Information

## References

[1] Mak KF, Lee C, Hone J, Shan J, Heinz TF (2010). Atomically thin *MoS*_2_: a new direct-gap semiconductor. Phys. Rev. Lett..

[2] Splendiani A (2010). Emerging photoluminescence in monolayer MoS_2_. Nano Lett..

[3] Mak KF, He K, Shan J, Heinz TF (2012). Control of valley polarization in monolayer MoS_2_ by optical helicity. Nat. Nanotechnol..

[4] Sallen G (2012). Robust optical emission polarization in MoS_2_ monolayers through selective valley excitation. Phys. Rev. B.

[5] Zeng H, Dai J, Yao W, Xiao D, Cui X (2012). Valley polarization in MoS_2_ monolayers by optical pumping. Nat. Nanotechnol..

[6] Cao T (2012). Valley-selective circular dichroism of monolayer molybdenum disulphide. Nat. Commun..

[7] Xiao D, Liu GB, Feng W, Xu X, Yao W (2012). Coupled spin and valley physics in monolayers of MoS_2_ and other group-VI dichalcogenides. Phys. Rev. Lett..

[8] Yin Z (2012). Single-layer MoS_2_ phototransistors. ACS Nano.

[9] Komsa HP, Krasheninnikov AV (2012). Effects of confinement and environment on the electronic structure and exciton binding energy of MoS_2_ from first principles. Phys. Rev. B.

[10] Feng J, Qian X, Huang CW, Li J (2012). Strain-engineered artificial atom as a broad-spectrum solar energy funnel. Nat. Photonics.

[11] Ben Amara I, Ben Salem E, Jaziri S (2016). Optoelectronic response and excitonic properties of monolayer MoS_2_. J. Appl. Phys..

[12] Mak KF (2013). Tightly bound trions in monolayer MoS_2_. Nat. Mater..

[13] Ross JS (2013). Electrical control of neutral and charged excitons in a monolayer semiconductor. Nat. Commun..

[14] Lui CH (2014). Trion-induced negative photoconductivity in monolayer *MoS*_2_. Phys. Rev. Lett..

[15] Rezk AR (2016). Acoustically-driven trion and exciton modulation in piezoelectric two-dimensional MoS_2_. Nano Lett..

[16] Zhang C, Wang H, Chan W, Manolatou C, Rana F (2014). Absorption of light by excitons and trions in monolayers of metal dichalcogenide MoS_2_: experiments and theory. Phys. Rev. B.

[17] Mouri S, Miyauchi Y, Matsuda K (2013). Tunable photoluminescence of monolayer MoS_2_ via chemical doping. Nano Lett..

[18] Singh A (2016). Trion formation dynamics in monolayer transition metal dichalcogenides. Phys. Rev. B.

[19] Scheuschner N (2014). Photoluminescence of freestanding single- and few-layer MoS_2_. Phys. Rev. B.

[20] Radisavljevic B, Radenovic A, Brivio J, Giacometti V, Kis A (2011). Single-layer MoS_2_ transistors. Nat. Nanotechnol..

[21] Ayari A, Cobas E, Ogundadegbe O, Fuhrer MS (2007). Realization and electrical characterization of ultrathin crystals of layered transition-metal dichalcogenides. J. Appl. Phys..

[22] Jones AM (2013). Optical generation of excitonic valley coherence in monolayer WSe2. Nat. Nanotechnol..

[23] Wang, Z., Shan, J. & Mak, K. F. Valley- and spin-polarized Landau levels in monolayer WSe2. *Nat. Nanotechnol*. 213 (2016). 10.1038/nnano.2016.213 27798606

[24] Wang, Z., Zhao, L., Mak, K. F. & Shan, J. Probing the spin-polarized electronic band structure in monolayer transition metal dichalcogenides by optical spectroscopy. *Nano Lett*. **17**, 740-746 (2017). 10.1021/acs.nanolett.6b03855 28103668

[25] Raja A (2017). Coulomb engineering of the bandgap and excitons in two-dimensional materials. Nat. Commun..

[26] Strinati G (1982). Dynamical shift and broadening of core excitons in semiconductors. Phys. Rev. Lett..

[27] Strinati G (1984). Effects of dynamical screening on resonances at inner-shell thresholds in semiconductors. Phys. Rev. B.

[28] Rösner M (2016). Two-dimensional heterojunctions from nonlocal manipulations of the interactions. Nano Lett..

[29] Ugeda MM (2014). Giant bandgap renormalization and excitonic effects in a monolayer transition metal dichalcogenide semiconductor. Nat. Mater..

[30] Bradley AJ (2015). Probing the role of interlayer coupling and coulomb interactions on electronic structure in few-layer MoSe_2_ nanostructures. Nano Lett..

[31] Andersen K, Latini S, Thygesen KS (2015). Dielectric genome of van der Waals heterostructures. Nano Lett..

[32] Ryou J, Kim YS, Santosh K, Cho K (2016). Monolayer MoS_2_ bandgap modulation by dielectric environments and tunable bandgap transistors. Sci. Rep..

[33] Esser A, Runge E, Zimmermann R, Langbein W (2000). Photoluminescence and radiative lifetime of trions in GaAs quantum wells. Phys. Rev. B.

[34] Narvaez GA, Bester G, Zunger A (2005). Excitons, biexcitons, and trions in self-assembled (In,Ga)AsGaAs quantum dots: recombination energies, polarization, and radiative lifetimes versus dot height. Phys. Rev. B.

[35] Machnikowski P, Kuhn T (2011). Nonlinear optical response of holetrion systems in quantum dots in tilted magnetic fields. Phys. Status Solidi C..

[36] Ganchev B, Drummond N, Aleiner I, Fal’ko V (2015). Three-particle complexes in two-dimensional semiconductors. Phys. Rev. Lett..

[37] Szyniszewski M, Mostaani E, Drummond ND, Fal’ko VI (2017). Binding energies of trions and biexcitons in two-dimensional semiconductors from diffusion quantum Monte Carlo calculations. Phys. Rev. B.

[38] Berkelbach TC, Hybertsen MS, Reichman DR (2013). Theory of neutral and charged excitons in monolayer transition metal dichalcogenides. Phys. Rev. B.

[39] Kylänpää I, Komsa HP (2015). Binding energies of exciton complexes in transition metal dichalcogenide monolayers and effect of dielectric environment. Phys. Rev. B.

[40] Deilmann T, Drüppel M, Rohlfing M (2016). Three-particle correlation from a many-body perspective: trions in a carbon nanotube. Phys. Rev. Lett..

[41] Jones AM (2016). Excitonic luminescence upconversion in a two-dimensional semiconductor. Nat. Phys..

[42] Boulesbaa A (2015). Observation of two distinct negative trions in tungsten disulfide monolayers. Phys. Rev. B.

[43] Yu H, Cui X, Xu X, Yao W (2015). Valley excitons in two-dimensional semiconductors. Natl. Sci. Rev..

[44] Xue J (2011). Scanning tunnelling microscopy and spectroscopy of ultra-flat graphene on hexagonal boron nitride. Nat. Mater..

[45] Geick R, Perry CH, Rupprecht G (1966). Normal modes in hexagonal boron nitride. Phys. Rev..

[46] Rohlfing M (2010). Electronic excitations from a perturbative LDA + *GdW* approach. Phys. Rev. B.

[47] Schmidt R (2016). Reversible uniaxial strain tuning in atomically thin Wse_2_. 2D Mater..

[48] Esat T (2016). A chemically driven quantum phase transition in a two-molecule Kondo system. Nat. Phys..

[49] Esat T (2015). Transfering spin into an extended *π* orbital of a large molecule. Phys. Rev. B.

[50] Neaton JB, Hybertsen MS, Louie SG (2006). Renormalization of molecular electronic levels at metal-molecule interfaces. Phys. Rev. Lett..

[51] Klein J (2016). Stark effect spectroscopy of mono-and few-layer MoS_2_. Nano Lett..

[52] Lippert S (2017). Influence of the substrate material on the optical properties of tungsten diselenide monolayers. 2D Mater..

[53] Bruix A (2016). Single-layer MoS_2_ on Au(111): band gap renormalization and substrate interaction. Phys. Rev. B.

[54] Hüser F, Olsen T, Thygesen KS (2013). Quasiparticle GW calculations for solids, molecules, and two-dimensional materials. Phys. Rev. B.

[55] Yan J, Jacobsen KW, Thygesen KS (2012). Optical properties of bulk semiconductors and graphene/boron nitride: the Bethe-Salpeter equation with derivative discontinuity-corrected density functional energies. Phys. Rev. B.

[56] Tanaka K (2005). Image charge effect on two-dimensional excitons in an inorganic-organic quantum-well crystal. Phys. Rev. B.

[57] Rohlfing M (2012). Redshift of excitons in carbon nanotubes caused by the environment polarizability. Phys. Rev. Lett..

[58] Soklaski R, Liang Y, Yang L (2014). Temperature effect on optical spectra of monolayer molybdenum disulfide. Appl. Phys. Lett..

[59] Zhang Y (2015). On valence-band splitting in layered MoS_2_. ACS Nano.

[60] Qiu DY, da Jornada FH, Louie SG (2015). Erratum: optical spectrum of MoS_2_: many-body effects and diversity of exciton states [Phys. Rev. Lett. **111**, 216805 (2013)]. *Phys. Rev. Lett.*.

[61] Onida G, Reining L, Rubio A (2002). Electronic excitations: density-functional versus many-body Green’s-function approaches. Rev. Mod. Phys..

[62] Rohlfing M, Louie SG (2000). Electron-hole excitations and optical spectra from first principles. Phys. Rev. B.

[63] Haydock R, Heine V, Kelly MJ (1972). Electronic structure based on the local atomic environment for tight-binding bands. J. Phys. C: Solid State Phys..

[64] Hernandez V, Roman JE, Vidal V (2005). SLEPc: a scalable and flexible toolkit for the solution of eigenvalue problems. ACM Trans. Math. Softw..

[65] Liang Y, Yang L (2015). Carrier plasmon induced nonlinear band gap renormalization in two-dimensional semiconductors. Phys. Rev. Lett..

