# Supplementary material for: Diversity of trion states and substrate effects in the optical properties of an MoS2 monolayer
Source: Nat Commun. 2017 Dec 14;8:2117. doi: 10.1038/s41467-017-02286-6 (PMC5730561; doi:10.1038/s41467-017-02286-6)
Supplement: Supplementary file 1 — Supplementary Information [file 41467_2017_2286_MOESM1_ESM.pdf]

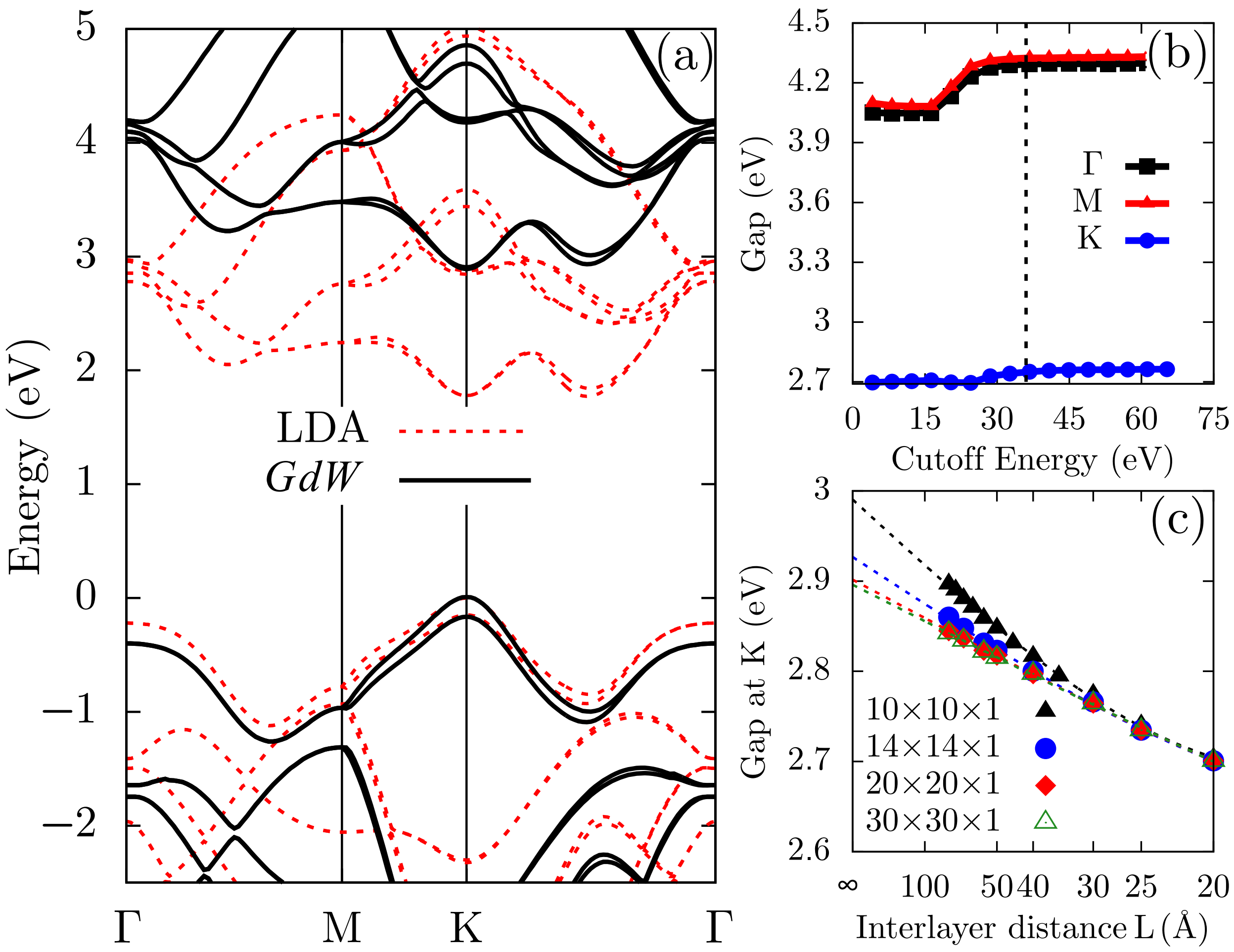

Supplementary Figure 1. Band structure of a MoS_2 monolayer. (a) LDA and *GdW* band structure comparison. For the LDA+*GdW* band structure a **k**-mesh of $30\times30\times1$ is employed. The interlayer distance is fixed at $L = 40$ Å (measured from one Mo to the next Mo in the neighboring supercell). To account for interlayer interaction, a scissors shift correcting the gap to the value for $L \to \infty$ is applied. An auxiliary basis of $2.5\,\mathrm{Ry} \approx 34\,\mathrm{eV}$ is used. (b) Convergence of the quasiparticle gap at three different points of the Brillouin zone. The dashed line indicates the used cutoff for (a). (c) Gap at the K point as a function of interlayer distance L for four different **k**-meshs.

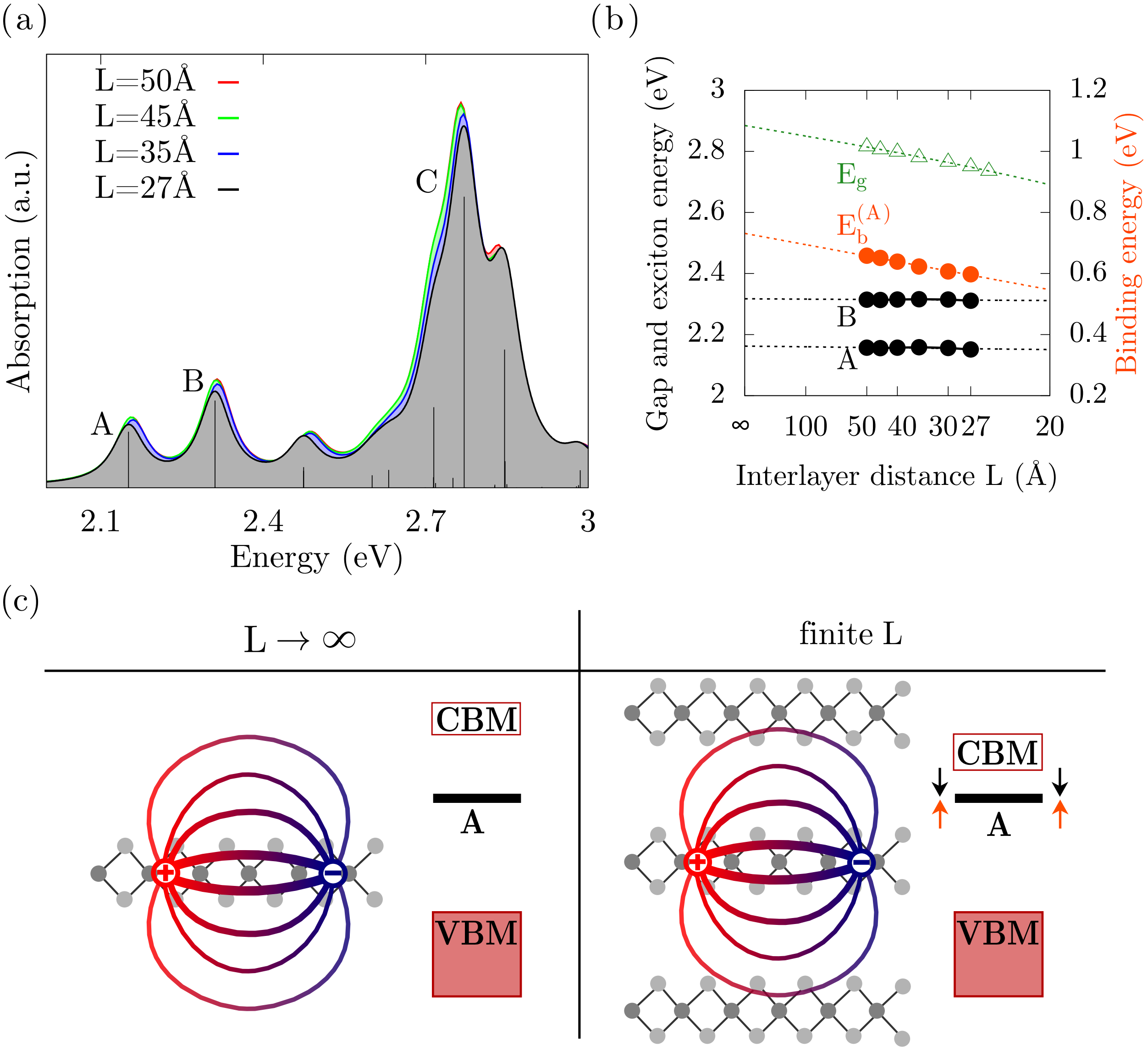

Supplementary Figure 2. Detailed exciton spectrum. (a) Exciton spectrum at four different distances to the neighboring layer, showing the A, B, and C excitons. Four valence and six conduction bands are included in the BSE-Hamilton operator and a **k**-mesh of $15 \times 15 \times 1$ is employed. An artificial broadening of 0.035 eV is used. (b) Band gap E_{g}, exciton binding energy $E_{\mathrm{b}}^{(\mathrm{A})}$ (orange), and excitation energies of the A and B excitons (black) as a function of interlayer distance L. (c) Left side: Isolated MoS_2 monolayer with an excited electron (red) -hole (blue) pair. Also given is a sketch of the energy positions involved (not to scale). Right side: When the supercell size is reduced, the CBM is shifted to lower energies, but the binding energy is also reduced, leaving the excitation energy (A) unchanged.

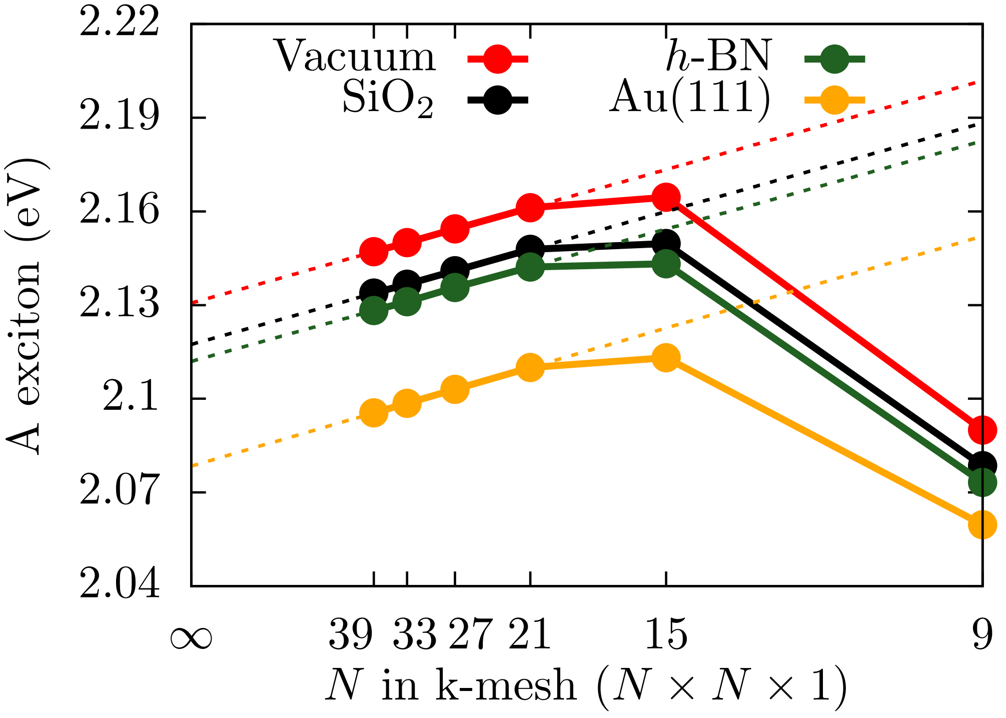

Supplementary Figure 3. Details of the A exciton. The figure shows the excitation energies of the A exciton as a function of the **k**-mesh for different substrates. The dashed line is a fit including all data point with $N \geq 21$ and shows the extrapolation to $N \to \infty$. Since these values are directly compared with trion calculations, two valence and four conduction bands are included and the interlayer distance is fixed to 45 Å.

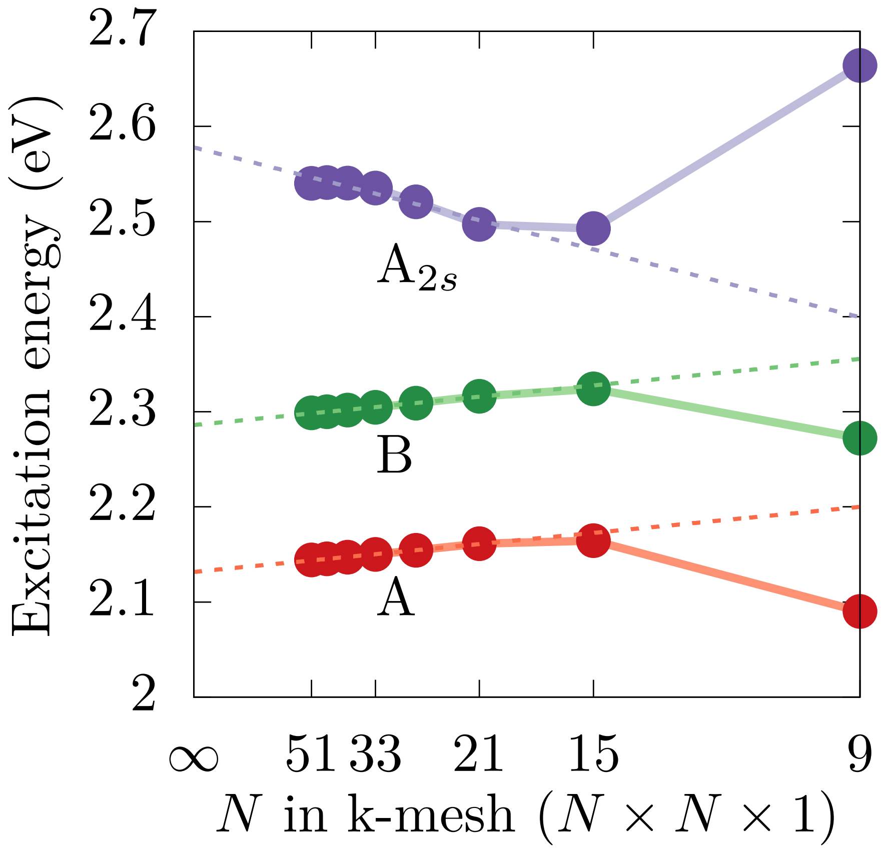

Supplementary Figure 4. Details of the A, B, and excited A_{2s} exciton. The figure shows the excitation energies of the A, B, and excited A_{2s} exciton in vacuum as a function of the **k**-mesh. The dashed line is a fit including all data point with $N \geq 21$ and shows the extrapolation to $N \to \infty$. The same parameters as for Supplementary Fig. 3 were used.

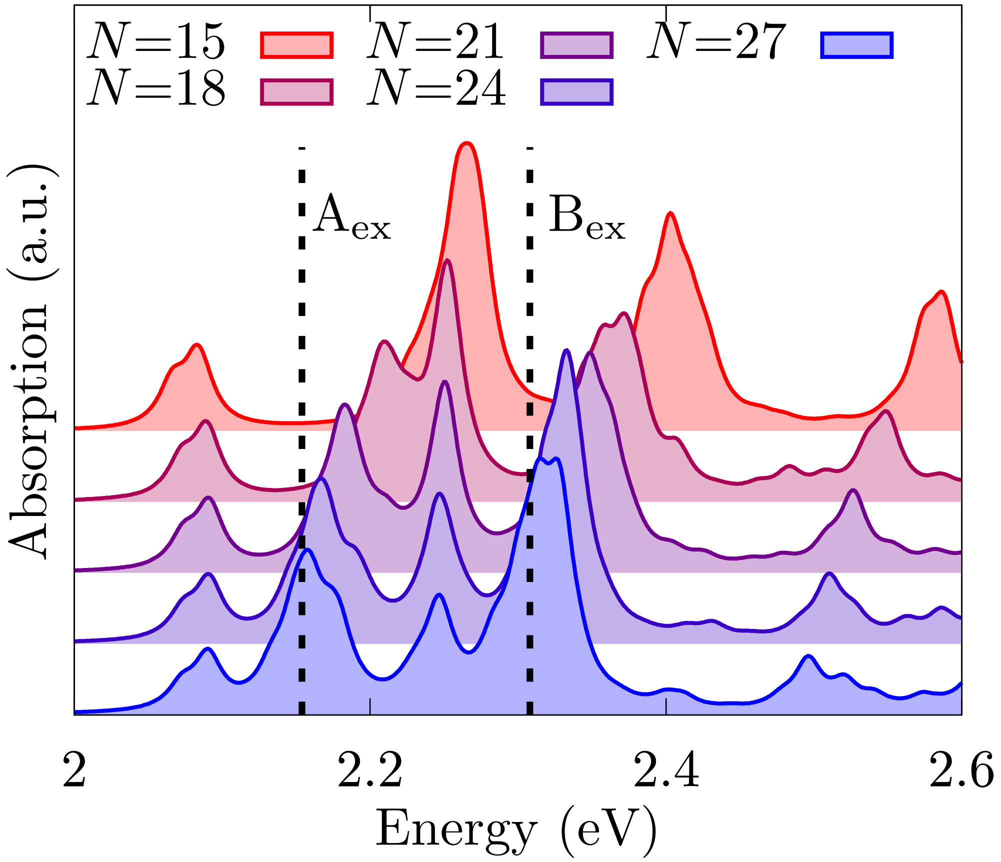

Supplementary Figure 5. Details of the trion calculations. The figure shows the convergence of the trion spectrum for different **k**-meshs $N \times N \times 1$. Since the two lowest conduction bands are only split by 15 meV, two possible positions of the additional electron are accounted for: the lowest CB and the CB+1 at K. The shown spectra already include both possibilities. An artificial broadening of 0.01 eV and two valence and four conduction bands are used. The spectrum is obtained by the Haydock recursion method. The dashed lines represent the excitation energies of the A and B excitons at $N = 27$. Different vertical offsets are used to improve visibility.

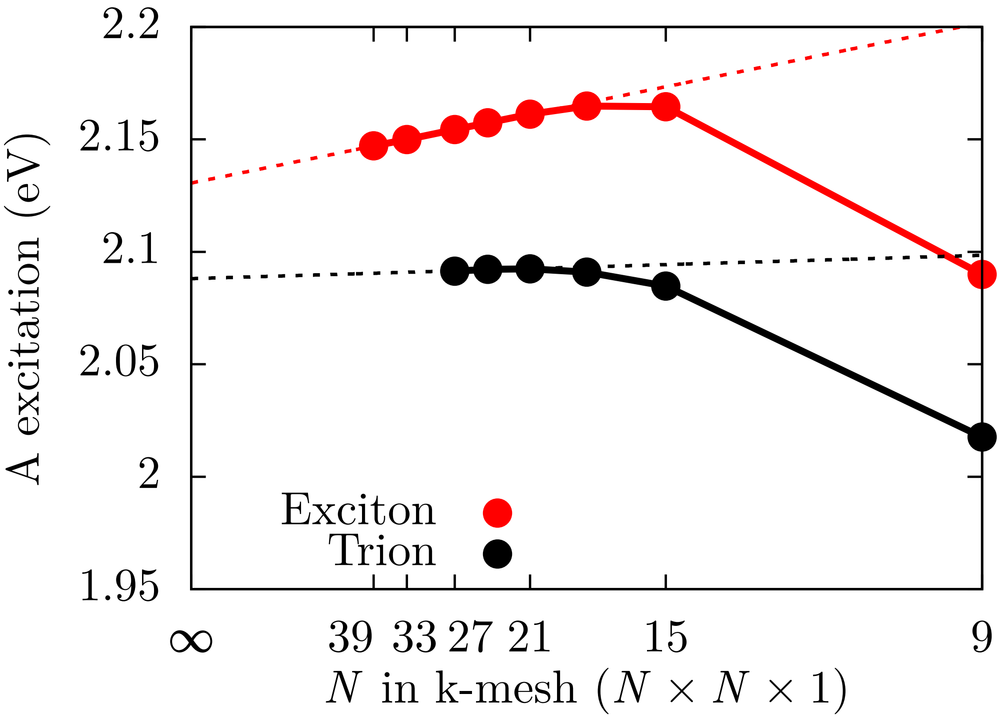

Supplementary Figure 6. Excitation energies of the A exciton and $A^-_{(1)}$ trion. The figure shows the excitation energies of the A exciton and $A^-_{(1)}$ trion (energetically lowest optically active trion) in vacuum for different **k**-meshs. The dotted line is a fit including all data point with $N \geq 21$ and shows the extrapolation to $N \to \infty$.

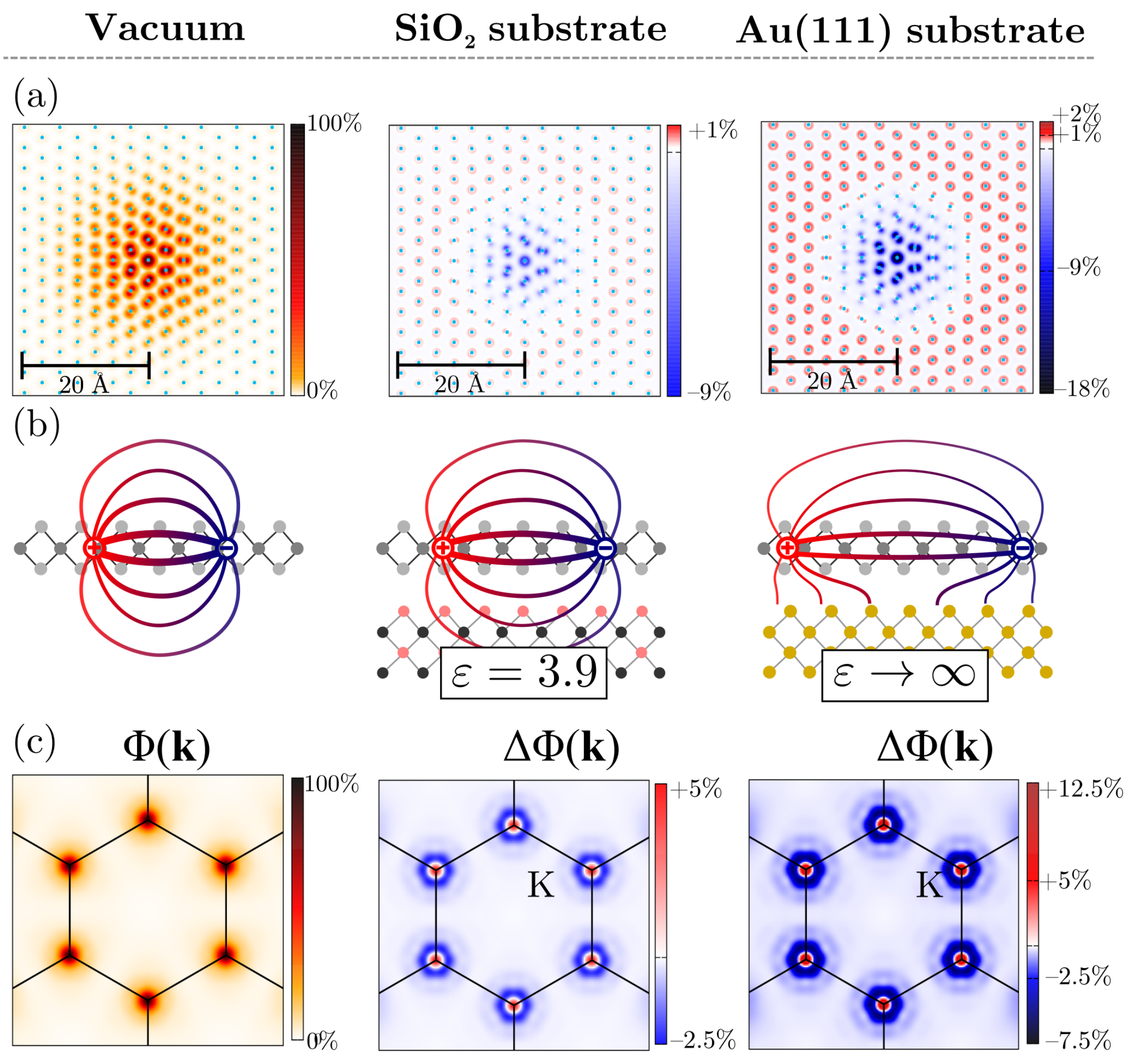

Supplementary Figure 7. Exciton wave function on different substrates. (a) Modulus squared of the exciton wave function of the A exciton in real space on the plane spanned by the Mo atoms (light blue points). For this, the position of the hole is fixed close to a Mo atom (at the maximum of the charge density of the valence band maximum at the K point). The corresponding root-mean-square radii are $\sqrt{\langle \mathbf{r}^2 \rangle} = 8.8/9.5/11.2$ Å for vacuum/SiO_2/Au(111), respectively. (b) Schematic of the exciton in vacuum, on SiO_2 and on Au(111): the stronger the screening, the larger the average distance between electron and hole. (c) Modulus of the contribution to the exciton in **k**-space $\Phi(\mathbf{k})$. For all substrate plots, the change in the distribution with respect to the vacuum situation is drawn, e.g., in **k**-space $\Delta\Phi(\mathbf{k}) = \Phi(\mathbf{k})_{\mathrm{sub}} - \Phi(\mathbf{k})_{\mathrm{vac}}$.

Supplementary Note 1: The LDA+*GdW* approximation

All results presented in the main text are obtained within the framework of many-body perturbation theory (MBPT), which allows a reliable, *ab initio* description of excited electronic states [1, 2]. We calculate the quasiparticle band structure starting from a density functional theory (DFT) calculation within the local density approximation (LDA) followed by the GW approach within the LDA+GdW approximation. The latter is numerically efficient and allows the inclusion of substrates. To handle trions, we use an extension of MBPT for excitons which includes a third excited particle. Both approaches are elaborated further followed by a careful analysis of the numerical stability of our results.

The LDA+GdW method is a first principles theoretical approach to the many-body physics of a given system. It states a further approximation in calculating the GW self-energy operator. Ref. [3] introduces it in detail showing its biggest advantage: numerical efficiency. Furthermore, it allows the straightforward inclusion of screening effects of substrates.

Starting point for most GW calculations are density functional theory energies and wave functions. They are used as input to calculate the self-energy $\Sigma = iGW$, which replaces the DFT-exchange correlation potential V_{xc} in the quasiparticle Hamiltonian

$$\hat{H}^{\mathrm{QP}}_{GW} = \hat{H}^{\mathrm{DFT}} + iGW - V_{\mathrm{xc}}. \tag{1}$$

The observation that motivates the LDA+GdW approach is that the usage of a hypothetical metallic screening approximately reproduces the DFT-LDA exchange correlation energy, i.e., $iGW_{\mathrm{metal}} \approx V_{\mathrm{xc}}$ (provided that iGW is a good approximation to Σ). This way, the construction of

$$\hat{H}^{\mathrm{QP}}_{\mathrm{LDA}+GdW} = \hat{H}^{\mathrm{DFT}} + iG(W - W_{\mathrm{metal}}) = \hat{H}^{\mathrm{DFT}} + iGdW \tag{2}$$

yields a good approximation for the quasiparticle Hamiltonian. Using Supplementary Eq. (2) to calculate the quasiparticle corrections states a huge advantage in efficiency, since now $\Delta\Sigma := iGdW$ (of the order of 1 eV) has to be calculated and converged instead of calculating V_{xc} and iGW separately (both often of the order of 10 eV) and then subtracting them. Within LDA+GdW we employ a second approximation: the usage of a model dielectric function (ε) for W and W_{metal}, which replaces the numerically demanding calculation of ε within, e.g., random-phase approximation. Due to the comparably small size of $iGdW$ (compared to iGW) the resulting error is tolerable, while such approximation would be more troublesome in full GW.

In the employed model, the dielectric function of the system is constructed by the contributions of the individual atoms. To simulate the screening of a substrate we only need to add the contributions of the substrate atoms to ε. Treated this way, the substrate only enters the calculation through the dielectric function, making it possible to get a clear picture of the changes induced by its screening only. We note that covalent bonding between the substrate and the monolayer is neglected. In principle, quantum-mechanical interaction of the electronic orbitals across the interface could add exciplex (or charge-transfer) contributions to the MoS_2 excitons, leading to red or blue shifts depending on orbital overlap (see Ref. [4] for similar effects in interacting carbon nanotubes). In practice, however, such effects would require perfect matching of the complex phase of the electronic states on both sides of the interface [4], which is already destroyed by the lattice mismatch between substrate and monolayer and can thus be considered as irrelevant.

To conduct a calculation, the unit cell of the substrate is stretched accordingly to fit the unit cell of the monolayer. The atomic polarizabilities were modified such that the substrate still has its natural dielectric constant. It was carefully checked that this distortion has a neglectable impact on the induced shifts due to screening. As explained by Rohlfing *et al.* [4], the shifts due to additional screening are not expected to depend strongly on details of the lattice structure. As mentioned in the main text, we use values for the dielectric constant ϵ_∞ of 3.9 (SiO_2 [5]), 4.95 / 4.10 ($\varepsilon_\infty^{\parallel}/\varepsilon_\infty^{\perp}$ *h*-BN [6]), and ∞ (Au).

We also note in passing that our current LDA+GdW approach, although being an additional approximation to the GW approach on an absolute energy scale, is ideally suited to address the red-shifts (i.e., differences) with sufficient accuracy [4].

Supplementary Note 2: Electron-hole excitations

Excitons are bound states of an excited electron and a hole. A well established framework for their theoretical description is given by the GW/BSE approach [7, 8]. Here, excitations are described through the Hamilton-matrix elements

$$\begin{aligned}\langle \mathbf{vc}|\hat{H}^{(eh)}|\mathbf{v'c'}\rangle =&(\epsilon_{\mathbf{c}} - \epsilon_{\mathbf{v}})\delta_{\mathbf{cc'}}\delta_{\mathbf{vv'}} && (\hat{H}_{\mathrm{BS}}) \\ &- (W_{\mathbf{v'c},\mathbf{vc'}} - V_{\mathbf{v'c},\mathbf{c'v}}) \, . && (\hat{H}_{eh})\end{aligned} \tag{3}$$

Where $|\mathbf{vc}\rangle := \hat{a}_{\mathbf{c}}^{\dagger}\hat{a}_{\mathbf{v}}|0\rangle$ denotes electron-hole pair excitations from the ground state of an

N-electron system $|0\rangle$. The index $\mathbf{v}$ ($\mathbf{c}$) refers to occupied valence (empty conduction) states. For a periodic system $\mathbf{v} = (v, \mathbf{k}_v)$ and $\mathbf{c} = (c, \mathbf{k}_c)$ combine the band index (v, c) with a wave number ($\mathbf{k}_v$, $\mathbf{k}_c$) for holes and electrons. The short-hand notation $\mathbf{v}$ ($\mathbf{c}$) is chosen for brevity. The Hamilton matrix has two contributions: the first is the bandstructure term $\hat{H}_{\mathrm{BS}}$ containing the quasiparticle energies $\epsilon_{\mathbf{c}}$, $\epsilon_{\mathbf{v}}$. The second is the electron-hole interaction $\hat{H}_{eh}$ which mixes the excitations and includes the bare Coulomb interaction $V_{\mathbf{ij},\mathbf{mn}} = \int \phi^*_{\mathbf{i}}(x)\phi^*_{\mathbf{j}}(y)V(x,y)\phi_{\mathbf{m}}(x)\phi_{\mathbf{n}}(y)dxdy$ with $V(x,x') = e^2/|\mathbf{r}-\mathbf{r}'|$ in the exchange term and the screened Coulomb interaction $W(\mathbf{r},\mathbf{r}') = \int \varepsilon^{-1}(\mathbf{r},\mathbf{r}'')V(\mathbf{r}'',\mathbf{r}')d^3r''$ in the direct term. Here, the coordinate $x = (\mathbf{r},\sigma)$ in the single particle states $\phi_{\mathbf{n}}(x)$ combines position and spin; correspondingly, the integration includes spin summation, i.e., $\int dx = \sum_{\sigma=\pm}\int d^3r$.

The diagonalization of Supplementary Eq. (3) yields the coupled electron-hole excitations of the system

$$|S,\mathbf{Q}\rangle = \sum_{\mathbf{vc}} B^{(S,\mathbf{Q})}_{\mathbf{vc}} |\mathbf{vc}\rangle \tag{4}$$

with the coefficients $B^{(S,\mathbf{Q})}_{\mathbf{vc}}$ and corresponding eigenvalues $\Omega^{(S,\mathbf{Q})}$ for an exciton with total momentum $\mathbf{Q}$. For the absorption of a photon with vanishing momentum $\mathbf{Q}=0$ the summation in Supplementary Eq. (4) over $\mathbf{k}_v$ and $\mathbf{k}_c$ is reduced to one sum with $\mathbf{k}_v = \mathbf{k}_c = \mathbf{k}$. Note that the discussion is restricted to the Tamm-Dancoff approximation.

Supplementary Note 3: Trion states

Trion states (also known as charged excitons) consist of three excited particles. Negatively charged states are formed from two electrons and one hole, while positively charged states are formed from one electron and two holes. In this section we focus on negatively charged states; the corresponding expressions for positive trions are analogous. A negative trion can be constructed as

$$|\mathbf{v}\mathbf{c}_1\mathbf{c}_2\rangle := \hat{a}^\dagger_{\mathbf{c}_2}\hat{a}^\dagger_{\mathbf{c}_1}\hat{a}_{\mathbf{v}}|0\rangle. \tag{5}$$

To avoid double counting, we require that $\mathbf{c}_1 < \mathbf{c}_2$, which assumes that the empty states are ordered. The definition of the order is arbitrary but has to be kept. Setting up similar

matrix elements as in Supplementary Eq. (3) yields

$$\langle \mathbf{v}\mathbf{c}_1\mathbf{c}_2|\hat{H}^{(eeh)}|\mathbf{v}'\mathbf{c}_1'\mathbf{c}_2'\rangle = \quad (6)$$

$$(\epsilon_{\mathbf{c}_1} + \epsilon_{\mathbf{c}_2} - \epsilon_{\mathbf{v}})\delta_{\mathbf{c}_1,\mathbf{c}_1'}\delta_{\mathbf{c}_2,\mathbf{c}_2'}\delta_{\mathbf{v},\mathbf{v}'} \quad (\hat{H}_{\mathrm{BS}})$$

$$+(W_{\mathbf{c}_1\mathbf{c}_2,\mathbf{c}_1'\mathbf{c}_2'} - W_{\mathbf{c}_1\mathbf{c}_2,\mathbf{c}_2'\mathbf{c}_1'})\delta_{\mathbf{v},\mathbf{v}'} \quad (\hat{H}_{ee})$$

$$-(W_{\mathbf{v}'\mathbf{c}_1,\mathbf{v}\mathbf{c}_1'} - V_{\mathbf{v}'\mathbf{c}_1,\mathbf{c}_1'\mathbf{v}})\delta_{\mathbf{c}_2,\mathbf{c}_2'} \quad (\hat{H}_{eh,1})$$

$$-(W_{\mathbf{v}'\mathbf{c}_2,\mathbf{v}\mathbf{c}_2'} - V_{\mathbf{v}'\mathbf{c}_2,\mathbf{c}_2'\mathbf{v}})\delta_{\mathbf{c}_1,\mathbf{c}_1'} \ . \quad (\hat{H}_{eh,2})$$

A detailed derivation of Supplementary Eq. (6) can be found in Ref. [9] and its Supplementary Material. The terms can be interpreted as follows: The first line of Supplementary Eq. (6) ($\hat{H}_{\mathrm{BS}}$) describes independent motion of each particle in the system's band structure, while the other terms describe the interaction (direct and exchange) between the two electrons ($\hat{H}_{ee}$), and between the hole and each of the electrons ($\hat{H}_{eh,1/2}$).

Diagonalization of the Hamiltonian set up by Supplementary Eq. (6) results in correlated trion states

$$|T,\mathbf{K}\rangle = \sum_{\mathbf{v}\mathbf{c}_1\mathbf{c}_2} A^{(T,\mathbf{K})}_{\mathbf{v}\mathbf{c}_1\mathbf{c}_2}|\mathbf{v}\mathbf{c}_1\mathbf{c}_2\rangle \ , \quad (7)$$

where the summation is restricted to $\mathbf{c}_1 < \mathbf{c}_2$. The total momentum of the trion is given by $\mathbf{K}$, which is constructed by the momenta of the electrons and the hole as $\mathbf{K} = \mathbf{k}_1 + \mathbf{k}_2 - \mathbf{k}_v$, which states a restriction to the sum in Supplementary Eq. (7). Note that the corresponding eigenvalues of Supplementary Eq. (7) denoted as $E^{(T,\mathbf{K})}$ are the energies of the trions. Optical transition energies found in an experimental spectrum would correspond to transitions from the trion state $|T,\mathbf{K}\rangle$ to a single excited electron state having the same momentum as the trion $|c\mathbf{K}\rangle$. Transition energies are thus given by

$$\Omega(|T,\mathbf{K}\rangle \leftrightarrow |c\mathbf{K}\rangle) = E^{(T,\mathbf{K})} - \epsilon_{c\mathbf{K}} \ . \quad (8)$$

Supplementary Note 4: Numerical results - band-structure calculations

Starting point for our many-body calculations is a DFT calculation carried out in the local density approximation (LDA), in the parametrization of Perdew and Zunger [10]. Norm-conserving pseudopotentials [11] in Kleinman-Bylander form [12] are used that also include spin-orbit interaction. We employ a basis of three shells of Gaussian orbitals with s, p, d

and s* symmetry and decay constants of [0.18, 0.49, 1.39] for Mo and [0.16, 0.56, 2.5] for S, all in units of a_B^{-2}. We find a theoretical lattice constant of 3.16 Å that coincides with the experimental value of bulk MoS_2 3.16 Å [13]. The position of the S atoms are structurally relaxed until forces are smaller than $10^{-4}\frac{\mathrm{Ry}}{a_\mathbf{B}}$. In reciprocal space a **k**-mesh of $10 \times 10 \times 1$ is employed.

All calculations are carried out using a code written by ourselves [14]. The resulting bandstructure is depicted in dashed red in Supplementary Fig. 1 (a) showing a direct band gap at K of 1.82 eV. The DFT calculation provides the single particle wave functions and energies that are used as input for the subsequent quasiparticle calculations.

As mentioned above, our MBPT calculations are carried out within the LDA+GdW approximation. The resulting band structure in direct comparison with the DFT bands is shown in Supplementary Fig. 1 (a). In MBPT the appearance of two-point functions requires a second, auxiliary basis. We use plane waves with a cutoff of 2.5 Ry $\approx$ 205 plane waves, which shows a convergence of the bands better than 0.05 eV as can be seen in Supplementary Fig. 1 (b). To account for interlayer interaction between different super cells, that lead to a closing of the electronic band gap, we calculate the electronic structure for multiple interlayer distances L (up to 80 Å), and extrapolate as L $\to \infty$ (see Supplementary Fig. 1 (c)). We note that this extrapolation scheme is only needed for the electronic properties, i.e., the gap and band structure, the absorption spectra are almost independent on the interlayer distance (see below). Supplementary Fig. 1 (c) also shows the **k**-mesh convergence of the quasiparticle gap, which is converged better than 0.05 eV for meshs greater than $14 \times 14 \times 1$.

Supplementary Note 5: Numerical results - BSE calculations

The excitonic properties are derived by solving Supplementary Eq. (7). Linearly polarized light (within the plane spanned by the monolayer) is used for all spectra. Supplementary Fig. 2 (a) shows the calculated BSE spectrum for different supercell sizes and thus different interlayer distances L. The spectrum shows the prominent A, B, and C excitons. Note that a **k**-mesh of $15 \times 15 \times 1$ is used to analyze the dependence on L. Most strikingly, the spectrum seems not to depend much on the interlayer distance (if L is large enough, i.e., $\gtrsim$ 27 Å). Supplementary Fig. 2 (c) schematically compares two situations: an isolated MoS_2 monolayer and a layer interacting with another one, i.e., for moderate L values. The excitation energy (A) is not shifted, while the band gap closes. This situation is similar

to the monolayer with and without a substrate. Two larger effects cancel each other: the band gap closes (Supplementary Fig. 2 (b), E_g green dots) and at the same time the binding energy is reduced ($E_\mathrm{b}^{(\mathrm{A})}$ orange dots), leaving the excitation energies almost constant (black dots, labeled A and B). We note that the same holds for the trion states as well.

For a comparison with trion spectra two valence and four conduction bands are used. Thereby the lower energy part of the spectrum, i.e., up to 2.6 eV is converged. Supplementary Fig. 3 shows the A exciton energy for different **k**-grids on different substrates. Note that the A excitons are already converged better than 0.05 eV at meshs as small as $15 \times 15 \times 1$, which we find equivalently for the B exciton. The excited A_{2s} state converges a bit slower, due to its more complicated spatial structure. The convergence of all three states is shown in Supplementary Fig. 4. This extremely fast convergence results from a combination of several aspects to our approach: In conventional GW calculations for d-electron systems, the s and p semicore electrons of the same shell (here: Mo $4s$+p) have to be included as valence electrons to incorporate their bare exchange interaction. Within LDA+GdW, on the other hand, the bare exchange interaction is not considered explicitly, and the semicore states can be treated as core states. This reduces the number of valence electrons (per Mo atom) from 14 to only 6. Moreover, we use identical **k**-meshs for the quasiparticle corrections and the electron-hole interaction, omitting the need for an interpolation scheme. The **k**-meshs for internal summation in the self-energy operator are also chosen exactly equivalent to the grids for the quasiparticle corrections. With such exactly matching meshs, the GW quasiparticle gap and the electron-hole interaction show the same asymptotic behavior with increasing mesh density, and the exciton energy (i.e., the combination of both) converges rapidly with the number of **k**-points. Additionally, the Coulomb interaction between adjacent supercells is not truncated, but exrapolated for an infinite interlayer distance for the electronic properties, which also reduces the convergence requirements [15].

Enhanced screening due to the substrate also affects the wave functions of the excited states. Due to stronger screening, the excitonic binding is weakened and the real space wave function gets further extended. This is illustrated in Supplementary Fig. 7 for the A exciton. The distribution of the electron in the plane spanned by the Mo atoms is shown in Supplementary Fig. 7 (a), assuming the hole is fixed close to the Mo atom in the center. The corresponding plots which include a substrate show how amplitude is shifted from the middle to the outer part of the electron distribution, thus extending the wave function in

real space. The Coulomb interaction binds the electron to the hole. Weakening this binding force via increased screening results in a larger electron distribution. The corresponding root-mean-square radii are $\sqrt{\langle \mathbf{r}^2 \rangle} = 8.8/9.5/11.2$ Å for vacuum/SiO_2/Au(111), respectively, where $\sqrt{\langle \mathbf{r}^2 \rangle} = \left(\frac{\langle S,\mathbf{Q}|(\mathbf{r}_h - \mathbf{r}_e)^2|S,\mathbf{Q}\rangle}{\langle S,\mathbf{Q}|S,\mathbf{Q}\rangle}\right)^{\frac{1}{2}}$ contains the distance between the hole and the electron $\mathbf{r}_h - \mathbf{r}_e$ and the A exciton wave function $|S, \mathbf{Q}\rangle$ evaluated in real space.

In reciprocal space this trend is inverted. Supplementary Fig. 7 (b) show how the contribution in $\mathbf{k}$-space of the A exciton gets more concentrated around K. The full width at half maximum for the vacuum situation ($0.116\,\frac{2\pi}{a}$) is reduced by 4.1% or 8.7% for the SiO_2 or Au(111) substrate, respectively.

Supplementary Note 6: Numerical results - Trion calculations

In the last step, one additional electron is included and bound negative trion states are calculated (the treatment of positive trions is fully analogous). This requires a diagonalization of the trion Hamilton-operator described by Supplementary Eq. (6). For our presented trion spectra the dimension is $\sim N_{\mathbf{k}}^2 N_{\mathrm{v}} N_{\mathrm{c}}^2 \sim 10^7$, using a grid of $N_{\mathbf{k}} = 27 \times 27 \times 1$ $\mathbf{k}$-points and two valence and four conduction bands (N_{v}, N_{c}), while the corresponding dimension of the BSE-Hamilton matrix would be merely $N_{\mathbf{k}} N_{\mathrm{v}} N_{\mathrm{c}} \approx 6000$. The size of the Hamilton operator puts a direct diagonalization out of reach. Fortunately, about 99.8 % of the matrix elements are zero, enabling an efficient route through recursive schemes like the Haydock recursion [16] when focusing on spectra, or the Lanczos algorithm or similar [17] to access eigenstates. It was carefully checked that both approaches results in almost identical spectra (not distinguishable by eye on the presented energy scales). Sufficient processors and memory were supplied by the HPC system PALMA of the University of Münster and the super-computer JURECA at Jülich Supercomputing Centre (JSC).

Independently from the BSE, the convergence of the trion spectrum with respect to the used $\mathbf{k}$-grid has to be carefully evaluated. The (Haydock-)spectra are shown in Supplementary Fig. 5. Not all trions converge at a similar rate, especially the first resonance shows a fast convergence, which becomes especially evident in Supplementary Fig. 6, which shows the energetic position of the A exciton and $\mathrm{A}^-_{(1)}$ trion in comparison, both extrapolated to $N \rightarrow \infty$, which yields a trion energy of 2.07 eV and thus a trion binding energy of 58 meV.

Supplementary References:

[1] Onida, G., Reining, L. & Rubio, A. Electronic excitations: density-functional versus many-body Green's-function approaches. *Rev. Mod. Phys.* **74**, 601–659 (2002). URL `http://link.aps.org/doi/10.1103/RevModPhys.74.601`.

[2] Rohlfing, M. & Louie, S. G. Electron-hole excitations and optical spectra from first principles. *Phys. Rev. B* **62**, 4927–4944 (2000). URL `http://link.aps.org/doi/10.1103/PhysRevB.62.4927`.

[3] Rohlfing, M. Electronic excitations from a perturbative LDA+*GdW* approach. *Phys. Rev. B* **82**, 205127 (2010). URL `http://link.aps.org/doi/10.1103/PhysRevB.82.205127`.

[4] Rohlfing, M. Redshift of Excitons in Carbon Nanotubes Caused by the Environment Polarizability. *Phys. Rev. Lett.* **108**, 087402 (2012). URL `http://link.aps.org/doi/10.1103/PhysRevLett.108.087402`.

[5] Xue, J. *et al.* Scanning tunnelling microscopy and spectroscopy of ultra-flat graphene on hexagonal boron nitride. *Nature materials* **10**, 282–285 (2011). URL `http://www.nature.com/nmat/journal/v10/n4/full/nmat2968.html`.

[6] Geick, R., Perry, C. H. & Rupprecht, G. Normal Modes in Hexagonal Boron Nitride. *Phys. Rev.* **146**, 543–547 (1966). URL `http://link.aps.org/doi/10.1103/PhysRev.146.543`.

[7] Strinati, G. Dynamical Shift and Broadening of Core Excitons in Semiconductors. *Phys. Rev. Lett.* **49**, 1519–1522 (1982). URL `http://link.aps.org/doi/10.1103/PhysRevLett.49.1519`.

[8] Strinati, G. Effects of dynamical screening on resonances at inner-shell thresholds in semiconductors. *Phys. Rev. B* **29**, 5718–5726 (1984). URL `http://link.aps.org/doi/10.1103/PhysRevB.29.5718`.

[9] Deilmann, T., Drüppel, M. & Rohlfing, M. Three-particle correlation from a Many-Body Perspective: Trions in a Carbon Nanotube. *Phys. Rev. Lett.* **116**, 196804 (2016). URL `http://link.aps.org/doi/10.1103/PhysRevLett.116.196804`.

[10] Perdew, J. P. & Zunger, A. Self-interaction correction to density-functional approximations for many-electron systems. *Phys. Rev. B* **23**, 5048–5079 (1981). URL `http://link.aps.org/doi/10.1103/PhysRevB.23.5048`.

[11] Hamann, D. R. Generalized norm-conserving pseudopotentials. *Phys. Rev. B* **40**, 2980–2987 (1989). URL http://link.aps.org/doi/10.1103/PhysRevB.40.2980.

[12] Kleinman, L. & Bylander, D. M. Efficacious Form for Model Pseudopotentials. *Phys. Rev. Lett.* **48**, 1425–1428 (1982). URL http://link.aps.org/doi/10.1103/PhysRevLett.48.1425.

[13] Böker, T. *et al.* Band structure of MoS_2, $MoSe_2$, and α-$MoTe_2$: Angle-resolved photoelectron spectroscopy and *ab initio* calculations. *Phys. Rev. B* **64**, 235305 (2001). URL http://link.aps.org/doi/10.1103/PhysRevB.64.235305.

[14] Rohlfing, M., Krüger, P. & Pollmann, J. Quasiparticle band-structure calculations for C, Si, Ge, GaAs, and SiC using Gaussian-orbital basis sets. *Phys. Rev. B* **48**, 17791–17805 (1993). URL http://link.aps.org/doi/10.1103/PhysRevB.48.17791.

[15] Hüser, F., Olsen, T. & Thygesen, K. S. How dielectric screening in two-dimensional crystals affects the convergence of excited-state calculations: Monolayer MoS_2. *Phys. Rev. B* **88**, 245309 (2013). URL http://journals.aps.org/prb/abstract/10.1103/PhysRevB.88.245309;http://arxiv.org/pdf/1311.1384.

[16] Haydock, R., Heine, V. & Kelly, M. J. Electronic structure based on the local atomic environment for tight-binding bands. *Journal of Physics C: Solid State Physics* **5**, 2845 (1972). URL http://stacks.iop.org/0022-3719/5/i=20/a=004.

[17] Hernandez, V., Roman, J. E. & Vidal, V. SLEPc: A Scalable and Flexible Toolkit for the Solution of Eigenvalue Problems. *ACM Trans. Math. Software* **31**, 351–362 (2005). URL http://dl.acm.org/citation.cfm?id=1089019.
